# Effect of local treatment in patients with oligo‐recurrence after surgery of distal bile duct cancer: A bi‐institutional study

**DOI:** 10.1002/cam4.5836

**Published:** 2023-03-23

**Authors:** So Jeong Yoon, Seung Soo Hong, Min Jae Gwon, Sang Hyun Shin, Jin Seok Heo, Chang Moo Kang, Kyung Sik Kim, Ho Kyoung Hwang, In Woong Han

**Affiliations:** ^1^ Division of Hepatobiliary‐Pancreatic Surgery, Department of Surgery, Samsung Medical Center Sungkyunkwan University School of Medicine Seoul South Korea; ^2^ Division of Hepatobiliary and Pancreatic Surgery, Department of Surgery, Severance Hospital Yonsei University College of Medicine Seoul South Korea

**Keywords:** bile duct cancer, cholangiocarcinoma, oligo‐recurrence, recurrence, survival

## Abstract

**Background:**

Distal extrahepatic bile duct (EHBD) cancer is highly recurrent. More than 50% of patients suffer from disease relapse after curative resection. Some patients present with oligo‐recurrence which could be a single loco‐regional mass or lesions limited to a single solid organ. The aim of this study was to examine the effect of local control (surgical resection or radiofrequency ablation) on survival outcomes in patients with oligo‐recurrent distal EHBD cancer.

**Methods:**

Data of 1219 patients who underwent surgery for distal EHBD cancer from 2000 to 2018 were retrospectively reviewed. Clinicopathological characteristics and survival outcomes of patients with recurrence were investigated. Post‐recurrence survival (PRS) was analyzed according to modalities of re‐treatment (local treatment or systemic therapy alone).

**Results:**

Among 654 patients with recurrence, 90 patients who had oligo‐recurrence showed better recurrence‐free and overall survival than patients with non‐oligo‐recurrent disease. Lymph node ratio and perineural invasion at initial pathology, timing of recurrence, and platelet‐to‐lymphocyte ratio at recurrence were independent risk factors for PRS in the oligo‐recurrent group. Patients with local treatment for oligo‐recurrence had better 3‐ and 5‐year PRS than those with systemic treatment alone (38.3% vs. 14.1%, *p* = 0.04; 28.3% vs. 7.1%, *p* = 0.04, respectively). Recurrence within 24 months after initial surgery was the only significant factor for PRS in the local treatment group.

**Conclusion:**

In patients with oligo‐recurrence after resection of distal EHBD cancer, post‐recurrence local treatment could improve survival outcomes, particularly for those with recurrence more than 2 years after initial resection.

## Graphical abstract

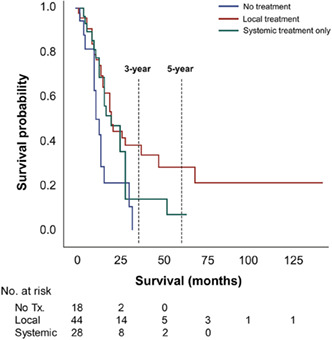

**Lay summary**

Distal bile duct cancer is highly recurrent, and chemotherapy remains the standard for recurrent cancer. However, there are some patients who present with a few lesions in a limited area, which can be considered as ‘oligo‐recurrence’. Oligo‐recurrent tumors can be removed by local treatment such as re‐operation or local ablative therapy. In the present study, we investigated the impact of local treatment on survival of patients with oligo‐recurrent bile duct cancer.

**Precis**

After resection of distal bile duct cancer, some patients present with oligo‐recurrent tumors, having potential for local treatment with curative intent. The present study identified that post‐recurrence local treatment improved survival in patients with oligo‐recurrent distal cholangicarcinoma, particularly for those with disease‐free interval of more than 2 years.

## 1 INTRODUCTION

Bile duct cancers, also known as cholangiocarcinomas, are highly lethal malignancies usually diagnosed at locally advanced stage with a low chance of curative surgery. Anatomically, distal extrahepatic bile duct (EHBD) cancer includes cholangiocarcinomas arising from the level of the cystic duct to distal common bile duct (CBD).^1^ EHBD cancers are also classified as mid and distal common bile duct (CBD) cancers. Surgical management of distal EHBD cancer can be segmental bile duct resection or pancreatoduodenectomy.^2^ Prognosis after surgical resection has been improved, with a recent study reporting that the 5‐year overall survival rate after R0 resection is 44%.^3^

It has been previously reported that more than half of patients suffer from recurrences after surgery.^4^ The pattern of recurrence could be either localized or diffused. The number and location of recurrent tumors can also vary according to previous studies.^5^, ^6^ Niibe et al have proposed the notion of oligo‐recurrence (a state of ≤5 metastatic or recurrent lesions after control of primary tumors).^7^ Our institution has previously explored oncologic benefits of re‐resection in recurrent cholangiocarcinoma and found that survival could be improved by surgery of loco‐regional oligo‐recurrent tumors.^6^ Some studies have reported the effectiveness of surgical interventions for recurrent biliary tract cancer.^8^, ^9^, ^10^, ^11^ However, most of these studies included other types of biliary tract cancers as well as distal EHBD cancer and were also based on limited numbers of patients.

As of now, only limited data are available on which patient might obtain survival benefit by local treatment of recurrent distal EHBD cancer and when to perform it. Therefore, the objectives of this study were: (1) to determine characteristics of patients with oligo‐recurrent distal EHBD cancer using a large cohort from bi‐institutional data; and (2) to investigate the effect and optimal timing of local re‐interventions for oligo‐recurrent tumors.

## 2 MATERIALS AND METHODS

### 2.1 Patient database

All consecutive patients with resected distal CBD cancer between January 2000 and December 2018 in Samsung Medical Center and Severance Hospital were eligible for this study. Data of 1219 patients were collected. All patients underwent either bile duct resection or pancreatoduodenectomy with regional lymph node dissection. Among them, patients who had recurrent disease during follow‐up were included in the analysis. Demographic characteristics and clinicopathological variables at initial surgery and at recurrence were retrospectively reviewed. Laboratory data included carbohydrate antigen 19–9 (CA 19–9), inflammatory markers such as neutrophil‐to‐lymphocyte ratio (NLR), and platelet‐to‐lymphocyte ratio (PLR). Pathology report included the size and differentiation of tumor, numbers of harvested and metastatic lymph nodes (LNs), lymphovascular invasion (LVI) or perineural invasion (PNI), and R status indicating curative resection. Lymph node ratio (LNR) was calculated as the number of metastatic LNs divided by the number of harvested LNs. This study was approved by the Institutional Review Boards of Samsung Medical Center (SMC 2021–07‐027) and Severance Hospital, Yonsei University College of Medicine (4–2022‐0929).

### 2.2 Diagnosis of recurrence and post‐recurrence treatment

Recurrence was diagnosed when patients presented with elevated CA 19–9 and suspicious tumors identified from imaging studies such as computed tomography (CT) scans or had pathologically proved as adenocarcinoma from biopsy. Positron emission tomography (PET) scan was additionally performed if the diagnosis was ambiguous. We defined oligo‐recurrence as a single localized recurrent lesion or no more than five multiple lesions in a single organ, which could be removed by local ablation or surgical resection. Post‐recurrence treatment plan was established considering characteristics of recurrence and general condition of a patient. For patients with oligo‐recurrence, re‐resection or radiofrequency ablation (RFA) was recommended. When patients had unbearable condition for general anesthesia or refused local treatment, additional chemotherapy or observation was considered.

### 2.3 Statistical analysis

Recurrence‐free survival (RFS) was calculated as the time interval between the date of operation and the date when recurrent tumors were identified in imaging studies. Overall survival (OS) was defined as the time from initial surgery to death from any causes. Post‐recurrence survival (PRS) was defined as the period from the date of recurrence to death.

Comparisons of peri‐operative clinical variables, intraoperative findings, postoperative outcomes, and pathological features were conducted using Student's *t*‐test and Chi‐squared test. Kaplan–Meier curves with log‐rank tests were used to compare survivals between patients with oligo‐recurrence and diffuse recurrence. Univariable and multivariable logistic regression models were used to identify factors associated with survival. Hazard ratios (HRs) were reported with 95% confidence intervals (CIs). The z‐test was performed to compare the survival probability at a specific time between patients. Variables with *p*‐values <0.05 were regarded as statistically significant. All statistical analyses were performed using IBM SPSS version 26 (SPSS Inc.) and SAS version 9.4 (SAS Institute Inc).

## 3 RESULTS

**FIGURE 1:**
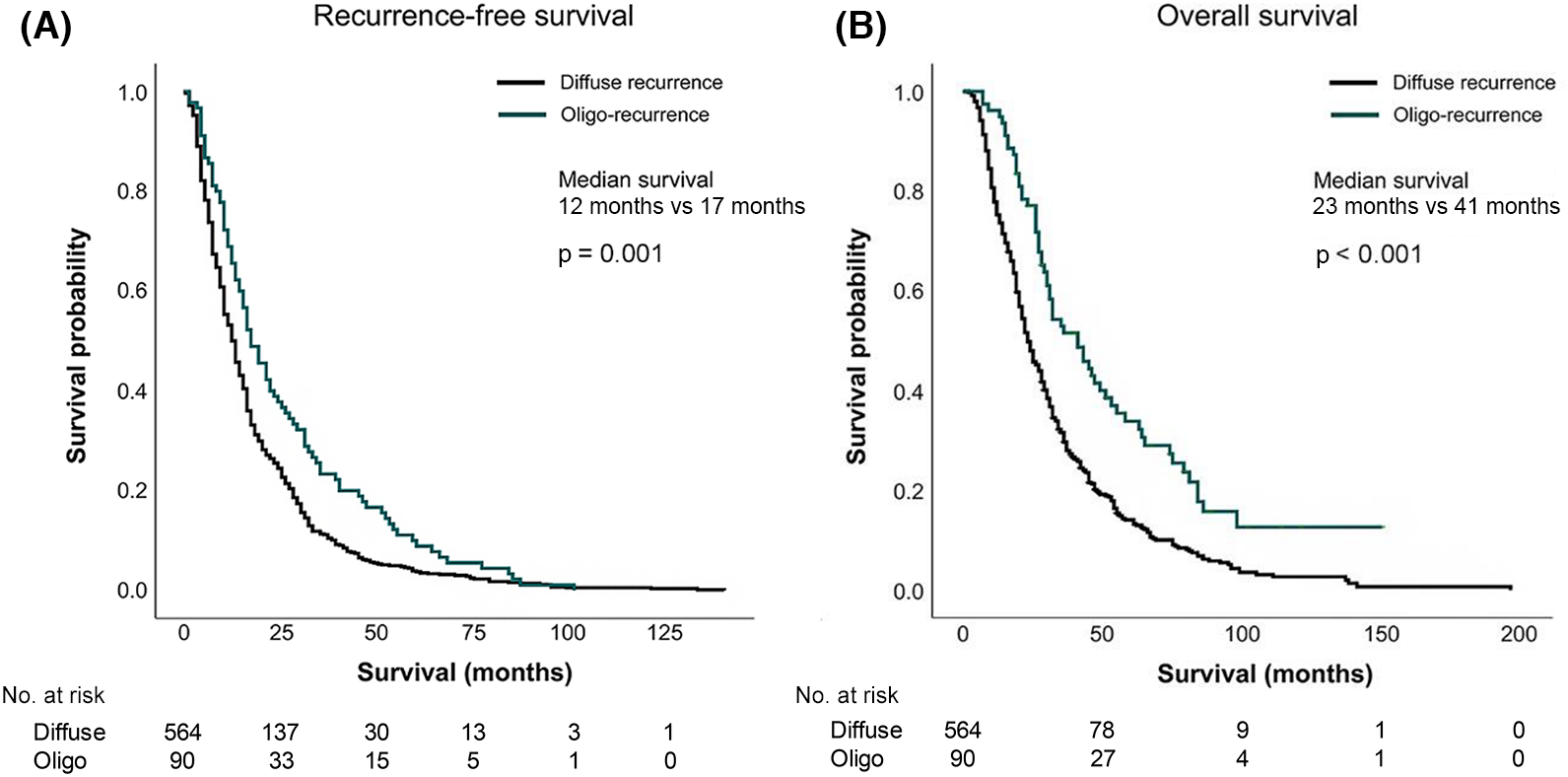
(A) Recurrence‐free survival and (B) Overall survival of patients with oligo‐recurrence and diffuse recurrence.

Among a total of 1219 patients with resected distal CBD cancer, 135 patients were lost to follow‐up and 654 (53.7%) patients showed recurrent cancer. The median time to recurrence was 13.0 months. Among the patients with recurrence, the number of patients with oligo‐recurrence was 90 (13.8%). Table 1 shows comparisons of clinicopathological characteristics between patients with oligo‐recurrence and diffuse recurrence. Mean body mass index (BMI) at initial operation was higher in patients with oligo‐recurrence (25.0 kg/m^2^ vs. 23.3 kg/m^2^, *p* = 0.011). Regarding pathological features, the numbers of both harvested and metastatic LNs were higher in the diffuse recurrence group (15.5 vs. 17.8, *p* = 0.047; 0.7 vs. 1.3, *p* = 0.004). There were more patients with LNR >0.1 in the diffuse recurrence group (24.3% vs. 15.6%, *p* = 0.045). LVI was more frequent in patients with diffuse recurrence than in those with oligo‐recurrence (53.7% vs. 37.9%, *p* = 0.017). In survival analysis, RFS and OS were significantly higher in the oligo‐recurrence group than in the diffuse recurrence group (median RFS: 17 months vs. 12 months, *p* = 0.001; median OS: 41 months vs. 23 months, *p* < 0.001) (Figure 1).

**TABLE 1:** Comparisons of clinicopathological characteristics between the oligo‐recurrence group and the diffuse recurrence group at initial operation.

Variables	Oligo‐recurrence group (*n* = 90)	Diffuse recurrence group (*n* = 564)	*p*
Age (year), mean	64.7 (±8.5)	66.0 (±8.7)	0.984
Sex, male	55 (61.1%)	372 (66.0%)	0.370
BMI (kg/m^2^), mean	25.0 (±14.3)	23.3 (±3.5)	0.011
Sx._Abdominal pain, yes	19 (21.1%)	10 (30.1%)	0.079
Sx._Jaundice, yes	52 (57.8%)	323 (57.3%)	0.928
Pre‐op CA19‐9 (U/mL), mean	914.0 (±6909.7)	814.7 (±5176.6)	0.628
Elevated CA19‐9, yes	46 (51.1%)	326 (59.4%)	0.140
Initial serum total bilirubin (mg/dL), mean	6.7 (±6.5)	7.6 (±7.6)	0.053
Pre‐op serum total bilirubin (mg/dL), mean	3.1 (±3.5)	3.3 (±3.5)	0.660
NLR, mean	2.8 (±2.1)	2.8 (±3.4)	0.994
PLR, mean	195.8 (±97.9)	180.0 (±96.2)	0.153
ASA score			0.966
I–II	55 (78.6%)	307 (79.7%)	
III–IV	15 (21.4%)	79 (20.4%)	
Operation type			0.155
BDR	28 (31.1%)	136 (24.1%)	
PD	62 (68.9%)	428 (75.9%)	
Combined vascular resection	2 (2.2%)	16 (2.8%)	0.741
Tumor size (cm), mean	2.5 (±1.1)	2.8 (±4.4)	0.536
Tumor differentiation			0.429
WD/MD	66 (76.7%)	383 (72.7%)	
PD/Undifferentiated	20 (23.3%)	144 (27.3%)	
LN metastasis, yes	29 (32.2%)	237 (42.5%)	0.067
No. of harvested LNs, mean	15.5 (± 9.3)	17.8 (±9.9)	0.047
No. of metastatic LNs, mean	0.7 (± 1.5)	1.3 (±2.4)	0.004
LN ratio >0.1	14 (15.6%)	141 (25.3%)	0.045
LVI, yes	25 (37.9%)	208 (53.7%)	0.017
PNI, yes	71 (85.5%)	428 (88.4%)	0.454
R0 resection	79 (87.8%)	497 (88.1%)	0.926
Major complication, yes	19 (21.1%)	102 (18.1%)	0.492
Adjuvant therapy, yes	42 (46.7%)	218 (38.9%)	0.164
Median recurrence‐free survival, months	17.0	12.0	0.001
Median overall survival, months	41.0	23.0	<0.001

Abbreviations: ASA, American Society of Anesthesiology; BDR, bile duct resection; BMI, body mass index; CA 19–9, carbohydrate antigen 19–9; LN, lymph node; LVI, lymphovascular invasion; MD, moderately differentiated; NLR, neutrophil‐to‐lymphocyte ratio; No., the number; PD (Operation type), pancreatoduodenectomy; PD (Tumor differentiation), poorly differentiated; PLR, platelet‐to‐lymphocyte ratio; PNI, perineural invasion; Pre‐op., preoperative; Sx., symptom; WD, well differentiated.

The most common site of oligo‐recurrence was the liver, followed by intra‐abdominal mass, remnant bile duct, and lung (Table 2). In the oligo‐recurrence group, 44 patients underwent post‐recurrence local treatment with or without additional systemic adjuvant therapy, 27 patients had systemic treatment alone, and 18 patients had no further treatment.

**TABLE 2:** Oligo‐recurrence sites and types of post‐recurrence treatment (*n* = 90).

Recur site	Liver (*n* = 56)	Remnant bile duct (*n* = 11)	Intra‐abdominal mass (*n* = 18)	Lung (*n* = 5)
Local tx.				
Re‐op. (*n* = 29)				
Without systemic tx.	5	3	6	4
With systemic tx.	3	4	4	0
RFA (*n* = 15)				
Without systemic tx.	Without systemic tx.	—	—	—
With systemic tx.	7	—	—	—
Systemic tx. Only (*n* = 27)	18	2	7	0
No tx. (*n* = 18)	15	2	1	1

Abbreviations: Re‐op., re‐operation; RFA, radiofrequency ablation; Tx., treatment.

**FIGURE 2:**
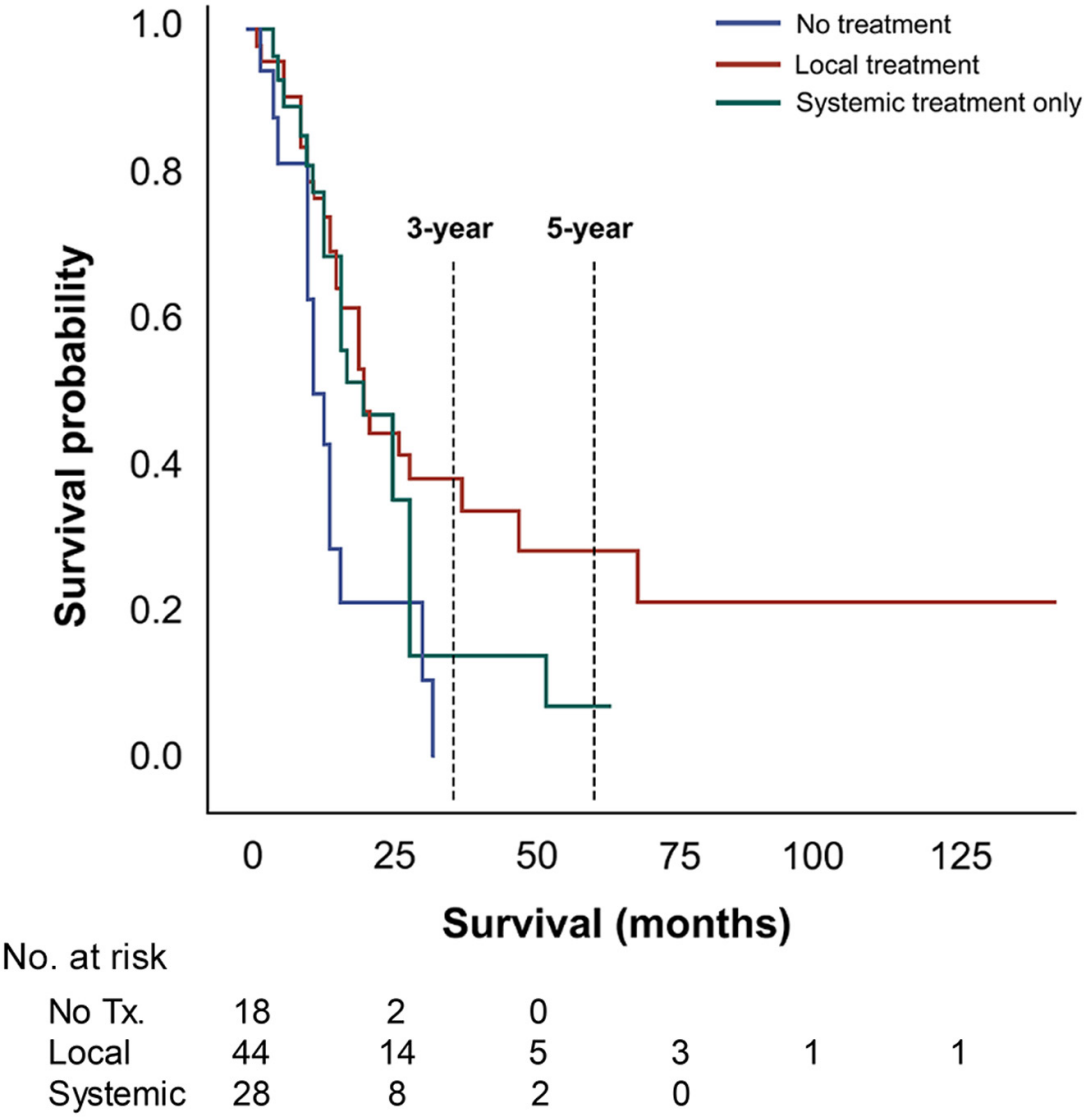
Post‐recurrence survival according to modalities of post‐recurrence treatment in patients with oligo‐recurrence.

Survival rates at 2, 3, and 5 years after recurrence were compared among patients with oligo‐recurrence (Figure 2). Three‐year and 5‐year PRS rates were significantly different between patients with local treatment and those with systemic treatment alone (3‐year PRS: 38.3% vs. 14.1%, *p* = 0.043; 5‐year PRS: 28.3% vs. 7.1%, *p* = 0.049). Risk factor analysis was performed to investigate factors related to PRS in the oligo‐recurrence group (Table 3). In multivariable analysis, LNR and PNI at initial pathology were statistically significant risk factors (HR: 15.037, 95% CI: 1.037–218.089, *p* = 0.047; HR: 4.287, 95% CI: 1.639–11.210, *p* = 0.003, respectively). Disease‐free interval of less than 24 months was associated with poor PRS (HR: 2.477, 95% CI: 1.312–4.674, *p* = 0.005). Among variables at the time of recurrence, PLR was an independent factor (HR: 1.007, 95% CI: 1.002–1.011, *p* = 0.006).

**TABLE 3:** Risk factor analysis for post‐recurrence survival in patients with oligo‐recurrence (*n* = 90).

Variable	Univariable analysis			Multivariable analysis		
	HR	95% CI	*p*	HR	95% CI	*p*
Clinicopathological factors at initial operation						
Age at operation	1.020	0.991–1.049	0.172			
Sex, female (ref. male)	1.176	0.692–1.998	0.548			
BMI	0.903	0.829–0.983	0.019	0.957	0.869–1.055	0.378
Sx._Abdominal pain, yes	0.930	0.492–1.756	0.822			
Sx._Jaundice, yes	1.054	0.625–1.778	0.843			
Elevated pre‐op. CA19‐9	1.185	0.707–1.988	0.520			
Initial serum total bilirubin	0.975	0.934–1.018	0.247			
Pre‐op. serum total bilirubin	0.941	0.846–1.047	0.266			
Pre‐op. NLR	0.992	0.893–1.102	0.876			
Pre‐op. PLR	1.000	0.998–1.003	0.741			
Operation type, PD (ref. BDR)	0.709	0.400–1.254	0.237			
Tumor size	0.952	0.782–1.160	0.628			
Tumor differentiation, PD (ref. WD/MD)	1.372	0.776–2.424	0.277			
LN metastasis, yes	1.400	0.823–2.381	0.215			
LN ratio	11.001	1.384–87.450	0.023	15.037	1.037–218.089	0.047
LVI, yes	1.136	0.603–2.142	0.693			
PNI, yes	1.968	0.882–4.393	0.098	4.287	1.639–11.210	0.003
R0 resection	1.308	0.659–2.594	0.443			
Adjuvant therapy, yes	0.813	0.487–1.357	0.428			
Disease‐free interval <24 months (*n* = 55)	1.827	1.038–3.215	0.037	2.477	1.312–4.674	0.005
Clinical factors at the timing of recurrence						
Age at recur	1.016	0.988–1.045	0.271			
BMI at recur	0.946	0.864–1.035	0.228			
Elevated CA 19–9 at recur	1.396	0.821–2.374	0.218			
Serum total bilirubin at recur	0.605	0.231–1.586	0.307			
NLR at recur	1.120	0.969–1.294	0.126			
PLR at recur	1.007	1.003–1.011	0.001	1.007	1.002–1.011	0.006
Recur site, liver (ref. others)	0.997	0.585–1.700	0.993			
Adjuvant chemotherapy after recur	0.665	0.399–1.109	0.118			

Abbreviations: BDR, bile duct resection; BMI, body mass index; CA 19–9, carbohydrate antigen 19–9; LN, lymph node; LVI, lymphovascular invasion; MD, moderately differentiated; NLR, neutrophil‐to‐lymphocyte ratio; PD (Op type), pancreatoduodenectomy; PD (Tumor differentiation), poorly differentiated; PLR, platelet‐to‐lymphocyte ratio; PNI, perineural invasion; Pre‐op., preoperative; Sx., symptom; WD, well differentiated.

**FIGURE 3:**
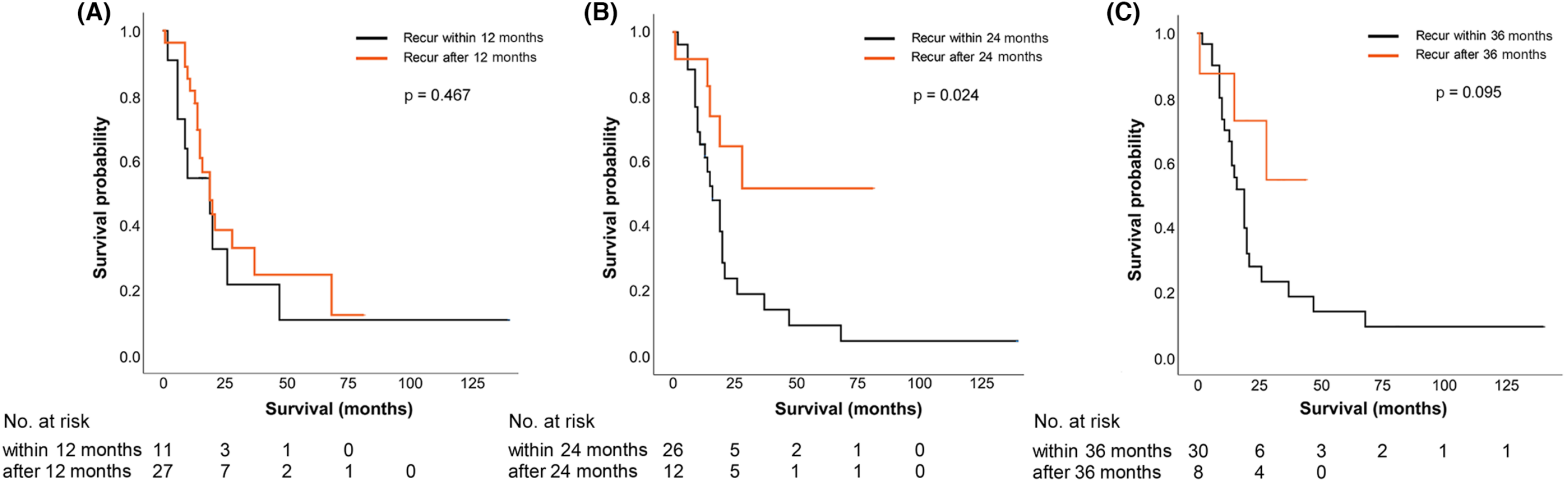
Post‐recurrence survival in patients with local treatment for oligo‐recurrence stratified by disease‐free interval of (A) 12 months, (B) 24 months, and (C) 36 months.

In the analysis for identifying relevant factors for PRS in the patients undergoing post‐recurrence local treatment, the only statistically significant factor was disease‐free interval (HR: 3.045, 95% CI: 1.042–8.895, *p* = 0.042) (Table 4). The PRS was compared between patient groups stratified by the disease‐free interval of 12, 24, and 36 months (Figure 3). The PRS differed significantly when the patients were divided over the disease‐free interval of 24 months (*p* = 0.024).

**TABLE 4:** Risk factor analysis for post‐recurrence survival in patients with local treatment for oligo‐recurrence (*n* = 44).

Variable	Univariable analysis					Multivariable analysis
	HR	95% CI	*p*	HR	95% CI	*p*
Clinicopathological factors at initial operation						
Age at operation	1.021	0.983–1.061	0.289			
Sex, female (ref. male)	1.114	0.505–2.461	0.789			
BMI	0.906	0.794–1.034	0.142			
Sx._Abdominal pain, yes	0.779	0.310–1.955	0.595			
Sx._Jaundice, yes	1.033	0.463–2.306	0.937			
Elevate pre‐op. CA19‐9	1.177	0.540–2.569	0.682			
Initial serum total bilirubin	0.976	0.918–1.036	0.420			
Pre‐op. serum total bilirubin	0.962	0.835–1.107	0.587			
Pre‐op. NLR	0.986	0.855–1.136	0.843			
Pre‐op. PLR	1.000	0.996–1.004	0.969			
Op type, PD (ref. BDR)	0.830	0.320–2.148	0.700			
Tumor size	1.005	0.793–1.274	0.964			
Tumor differentiation, PD (ref. WD/MD)	1.627	0.680–3.890	0.274			
LN metastasis, yes	1.308	0.574–2.979	0.522			
LN ratio	132.23	0.042–419,432	0.235			
LVI, yes	1.111	0.427–2.891	0.829			
PNI, yes	1.588	0.533–4.730	0.406			
R0 resection	1.433	0.484–4.240	0.516			
Adjuvant therapy, yes	0.698	0.311–1.564	0.382			
Disease‐free interval <24 months (*n* = 30)	2.871	1.079–7.637	0.035	3.045	1.042–8.895	0.042
Clinical factors at the timing of recurrence						
Age at recur	1.015	0.976–1.054	0.459			
BMI at recur	0.977	0.863–1.106	0.715			
Elevated CA 19–9 at recur	1.950	0.881–4.319	0.100			
Serum total bilirubin at recur	0.559	0.164–1.903	0.352			
NLR at recur	1.075	0.817–1.414	0.605			
PLR at recur	1.007	1.000–1.015	0.060	1.006	0.997–1.015	0.192
Adjuvant (systemic) treatment after recur	0.706	0.326–1.533	0.379			

Abbreviations: BDR, bile duct resection; BMI, body mass index; CA 19–9, carbohydrate antigen 19–9; LN, lymph node; LVI, lymphovascular invasion; MD, moderately differentiated; NLR, neutrophil‐to‐lymphocyte ratio; PD (Op type), pancreatoduodenectomy; PD (Tumor differentiation), poorly differentiated; PLR, platelet‐to‐lymphocyte ratio; PNI, perineural invasion; Pre‐op., preoperative; Sx., symptom; WD, well differentiated.

## 4 DISCUSSION

Cholangiocarcinomas have an aggressive behavior. Relapses are common even after curative resection. Patients with recurrent disease have been treated in a palliative manner, usually with systemic chemotherapy or supportive care only.^12^ In recent years, a number of studies have reported that local control of recurrent tumors such as re‐resection or ablative therapy might improve survival outcomes.^8^, ^9^, ^10^, ^11^ However, most of the previous studies included only intrahepatic cholangiocarcinoma. Only a limited number of studies have been conducted on distal CBD cancer. Considering that biological, clinicopathological features, and prognosis vary among anatomically classified cholangiocarcinomas,^13^ an in‐depth investigation on recurrent distal CBD cancer as a single disease entity is necessary.

Regarding oligo‐recurrence, there have been previous reports on isolated recurrence of biliary tract or pancreatic cancer after resection, even before the concept was proposed in 2010.^7^ In terms of pancreatic cancer, it is known that approximately 30% of patients with local recurrence have isolated recurrence.^14^ According to a recent meta‐analysis,^15^ re‐resection of isolated recurrent pancreatic cancer could offer survival benefit. Furthermore, it has been reported that among patients with potentially curative recurrent lesions, those with a single pulmonary recurrence with a longer disease‐free interval are more likely to have a better chance of survival.^16^ Similarly, there are a few case series from high‐volume centers reporting reoperation for recurrent distal bile duct cancer,^6^, ^8^, ^10^ with the authors arguing the feasibility of resecting recurrent tumors. However, little is known about the selection of eligible patients and the proper timing of re‐treatment. Therefore, our study placed more emphasis on patients with oligo‐recurrence of distal CBD cancer, aiming to explore any distinct features of them and to investigate proper candidates who might benefit from post‐recurrence local treatment.

Some risk factors related to survival after resection of distal CBD cancer have been reported, including tumor differentiation, LN metastasis, resection margin, PNI, and depth of invasion.^17^ In the present study, patients with oligo‐recurrence had better survival outcomes with lower LNR and less LVI than those with non‐oligo‐recurrence. In the oligo‐recurrent group, LNR, PNI at initial pathology, and PLR at recurrence were associated with PRS. It is noticeable that LNR, rather than the presence of LNM itself, was an independent prognostic factor, which is consistent with the result of a former study.^18^ Since LNR reflects the number of retrieved LNs as well as the number of metastatic LNs, the number of LN yielded could be influential. A recent study has suggested that at least five nodes should be examined to apply the eighth edition of the American Joint Committee on Cancer (AJCC) system with accuracy.^19^ In order to utilize LNR as a prognosticator, a further study on the appropriate number of lymph node yield and the optimal cut‐off value is necessary.

Inflammatory markers have recently emerged as potential prognostic factors for several malignancies including biliary tract cancers.^20^ The possible relationship between inflammatory markers and oncologic outcomes arises from the role of chronic inflammation in developing cancers. According to some previous studies, systemic inflammation could be either cause or effect of malignancy.^21^, ^22^ Regardless, inappropriately activated immune system might result in increases of circulating neutrophil and platelet. Hence, NLR and PLR could reflect biological activity of tumor. Kitano et al have reported that elevated preoperative PLR is associated with poor survival outcomes in patients with resected extrahepatic cholangiocarcinoma.^23^ In the present study, PLR at recurrence, rather than preoperative value, was an independent risk factor for PRS. It might suggest that PLR at recurrence reflects the nature of recurrent tumor, not primarily resected tumor, and affects survival after recurrence. Many other inflammatory and immune‐nutritional markers such as lymphocyte‐to‐monocyte ratio, Glasgow prognostic score, and prognostic nutritional index are under investigation. Considering that data of these markers are readily available from perioperative blood tests, additional research on clinical implication and prognostic value of the abovementioned markers should be undertaken.

With regard to the timing of post‐recurrence treatment, based on disease‐free interval of 24 months, PRS was found to be significantly different among patients with local treatment. Furthermore, the only predictive factor for PRS in this group was RFS of 24 months. This result is somewhat consistent with a study by Takahashi et al, which reported that survival after re‐resection of recurrent biliary tract cancer was better in patients with disease‐free interval of two or more years.^10^ This can be also associated with the issue of early or late recurrence. In this respect, patients with late recurrence might get a better chance of survival by post‐recurrence treatment. A few studies have investigated early recurrence of distal CBD cancer after surgery. One of them has defined early recurrence as disease relapse within 1 year after curative resection, and reported that lymphatic invasion is a significant prognosticator for early recurrence.^24^ Another study has found that older age, elevated serum CA 19–9, and PNI are associated with recurrence of EHBD cancer within 6 months of surgery.^25^

To select patients who have a high possibility of prolonged survival by post‐recurrence local treatment, further work is needed to understand implications of the timing of recurrence and to explore risk factors related to better outcomes of recurrent distal CBD cancer.

This study has a number of potential limitations that need to be considered. First, significant biases might have affected results owing to the retrospective design. Among all patients who underwent surgery, some patients who were lost to follow‐up without information on recurrence were excluded. Also, due to its nature of multi‐center study, there existed a few differences in detail between institutions regarding post‐operative patient follow‐up and decision on post‐recurrence treatment. As for patients with oligo‐recurrence, decision on local treatment was shared with patients considering their overall conditions. However, no objective indicator was available for determining if a patient could tolerate local therapy. There were no criteria for selecting modalities of local treatment between RFA and surgical resection. This led to unsolved issue regarding which modalities would have affected the PRS in the patients with local treatment. Despite all these limitations, we obtained comprehensive results for understanding oligo‐recurrence of distal EHBD cancer and the effect of post‐recurrence local treatment by analyzing a large cohort from various angles. Accordingly, the present study demonstrates that local therapy for late‐onset oligo‐recurrent distal CBD cancer might offer a patient a better chance of survival on a long‐term basis. Further prospective studies are needed to verify appropriate candidates and the feasibility of local treatment for recurred distal CBD cancer.

## AUTHOR CONTRIBUTIONS

**So Jeong Yoon:** Conceptualization (lead); data curation (lead); formal analysis (lead); writing – original draft (lead); writing – review and editing (lead). **Seung Soo Hong:** Conceptualization (lead); data curation (lead); formal analysis (lead); writing – original draft (lead); writing – review and editing (lead). **Min Jae Gwon:** Formal analysis (equal); writing – original draft (equal); writing – review and editing (equal). **Sang Hyun Shin:** Conceptualization (equal); data curation (equal); formal analysis (equal); writing – original draft (equal); writing – review and editing (equal). **Jin Seok Heo:** Conceptualization (equal); data curation (equal); formal analysis (equal); writing – original draft (equal); writing – review and editing (equal). **Chang Moo Kang:** Conceptualization (equal); data curation (equal); formal analysis (equal); writing – review and editing (equal). **Kyung Sik Kim:** Conceptualization (equal); data curation (equal); formal analysis (equal); writing – review and editing (equal). **Ho Kyoung Hwang:** Conceptualization (lead); data curation (lead); formal analysis (lead); writing – original draft (lead); writing – review and editing (lead). **In Woong Han:** Conceptualization (lead); data curation (lead); formal analysis (lead); writing – original draft (lead); writing – review and editing (lead).

## FUNDING INFORMATION

This work was supported by the National Research Foundation of Korea grant funded by the Korea government (Ministry of Science and ICT) (NRF‐2022M3H4A1A01012816).

## CONFLICT OF INTEREST STATEMENT

The authors have no conflicts of interest relevant to this study to disclose.

## Data Availability

The data that support the findings of this study are available on request from the corresponding author. The data are not publicly available due to privacy or ethical restrictions.
